# Mammalian mesenchymal stromal cells enhance zebrafish fin regeneration

**DOI:** 10.1186/s13619-025-00273-7

**Published:** 2026-01-15

**Authors:** Dora Sapède, Claudia Terraza-Aguirre, Jholy De La Cruz, Claire Vinatier, Jérôme Guicheux, Christian Jorgensen, Farida Djouad

**Affiliations:** 1 IRMB, Université de Montpellier, INSERM, Montpellier, France; 2 CHU Montpellier, Montpellier, France; 3 Nantes Université, Oniris, CHU Nantes, INSERM, Regenerative Medicine and Skeleton, RMeS, UMR 1229, Nantes, France

**Keywords:** Mesenchymal stromal cells, Macrophages, PGE2, Regeneration, Zebrafish

## Abstract

Mesenchymal stromal cells (MSCs) possess well-described immunoregulatory properties, yet their capacity to drive regeneration in vertebrates is still debated and their mechanisms of action remain to be fully elucidated. In this study, we used zebrafish larvae, a highly regenerative vertebrate model to study the effects of MSC delivery on caudal fin fold regeneration and monitored macrophage dynamics through live imaging in fluorescent reporter lines. We found that MSCs enhanced fin regeneration by increasing the early recruitment of inflammatory (*tnfa* +) macrophages at 1-day-post-amputation (dpA), and accelerating resolution between 2 and 3 dpA. Given the established role of prostaglandin E2 (PGE2) in MSC-mediated immunoregulation, we examined its contribution using indomethacin, a cyclooxygenase inhibitor that suppresses PGE2 production in grafted MSCs. We observed that PGE2 inhibition abolished the pro-regenerative effect of MSCs and maintained elevated tnfa + macrophage levels. PGE2-inhibited MSCs were more susceptible to phagocytosis by both zebrafish and mammalian macrophages, while maintaining viability, indicating a loss of PGE2-mediated protection in treated cells. Together, these findings demonstrate that MSC-derived PGE2 is essential for MSC regenerative function by promoting MSC persistence and modulating macrophage behavior, highlight the zebrafish as a powerful in vivo platform to dissect stem cell–immune interactions and optimize MSC-based regenerative strategies.

**Supplementary Information:**

The online version contains supplementary material available at 10.1186/s13619-025-00273-7.

## Background

Mesenchymal stromal cells (MSCs) are multipotent cells known for their ability to differentiate into various cell types and for their remarkable immunomodulatory and tissue repair properties. Their capacity to promote tissue healing and repair has drawn significant attention in regenerative medicine, particularly in contexts such as skin burns, diabetic ulcers, and radiation-induced injuries (Jo et al. 2021; Maranda et al. 2017; Rodgers and Jadhav 2018; Zhao et al. 2023). These studies have emphasized the ability of MSCs to reduce inflammation, promote cell proliferation, and facilitate tissue repair, mainly by secreting trophic factors such as cytokines and growth factors. However, their regenerative properties have never been demonstrated.

Epimorphic regeneration is the process by which lost structures are replaced by a functional replica through proliferation and differentiation of progenitor cells (Laplace-Builhe et al. 2020). In mammals, epimorphic regeneration is scarce, the digit tip of mice, primates, and humans being the only part of the limb capable of regenerating at all developmental stages, including adulthood (for review, see Simkin et al. 2015). Following digit tip amputation, a blastema forms, restoring bone, nerves, and skin, with the nail organ playing a crucial role in this regeneration (Lehoczky and Tabin 2015). Despite a wealth of preclinical and clinical evidence supporting their therapeutic potential, the role of MSCs in more complex processes, such as epimorphic regeneration of tissues, remains largely unexplored.

The zebrafish (*Danio rerio*) is one of the most powerful vertebrate models for studying epimorphic regeneration, renowned for its remarkable efficiency and limitless potential in regenerating its caudal fin (Azevedo et al. 2011; Pfefferli and Jazwinska 2015). The caudal fin fold in zebrafish larvae features a simple structure, consisting of epithelial layers and mesenchymal cells which start depositing collagen fibers that will later form an exoskeleton of rays made of intramembranous bone. At larval stages, this model system provides additional advantages, including simplicity with a reduced number of cells and cell types, rapid regrowth upon severe amputation (Kawakami et al. 2004), body transparency for live imaging and a proficient set of genetic and molecular tools for manipulating and identifying the mechanisms involved in these biological processes.

Although MSCs are widely recognized for their role in wound healing or tissue repair, their involvement in the context of complex tissue regeneration raises several unanswered questions. Do MSCs act directly by differentiating into specific cell types and contributing to tissue replacement? Or do they exert their effects indirectly by modulating the regenerative microenvironment through the secretion of trophic factors, or exosomes, which are known to influence local cellular proliferation, migration, and differentiation (Caplan and Correa 2011; Vizoso et al. 2017). An emerging hypothesis is that MSCs interact with host immune cells, particularly with macrophages, to influence the outcome of regenerative processes, either by modulating their recruitment, activation, or polarization (e.g., anti- versus pro-inflammatory phenotypes) (Bernardo and Fibbe 2013) or by modifying macrophage phagocytic activity, a mechanism that could play a pivotal role in resolving inflammation and promoting regeneration (Wang et al. 2014).

Building on our previous findings that epimorphic regeneration in zebrafish larvae relies on the precise recruitment and activation of macrophage subtypes at the wound site (Nguyen-Chi et al. 2017), this study aims to investigate the impact of MSC administration on the immune response following fin fold amputation and throughout regeneration. Using the zebrafish model, this study clarifies the contributions of MSCs to tissue regeneration. Specifically, we describe their effects on caudal fin regeneration, their influence on macrophage kinetics of recruitment and activation, and their capacity to modulate immune cell phagocytic activity.

Among the numerous soluble mediators secreted by MSCs, prostaglandin E2 (PGE2) has been described as a critical factor mediating their immunomodulatory and protective functions (Kulesza et al. 2023; Pinzariu et al. 2025). Although these effects in mammalian models have been extensively documented, the contribution of MSC-derived PGE2 to vertebrate epimorphic regeneration remains unexplored. Zebrafish larvae, with their remarkable regenerative efficiency and suitability for in vivo imaging, provide an ideal system to address this question.

## Results

### Exogenous MSCs enhance caudal fin regeneration in zebrafish

**Fig. 1:**
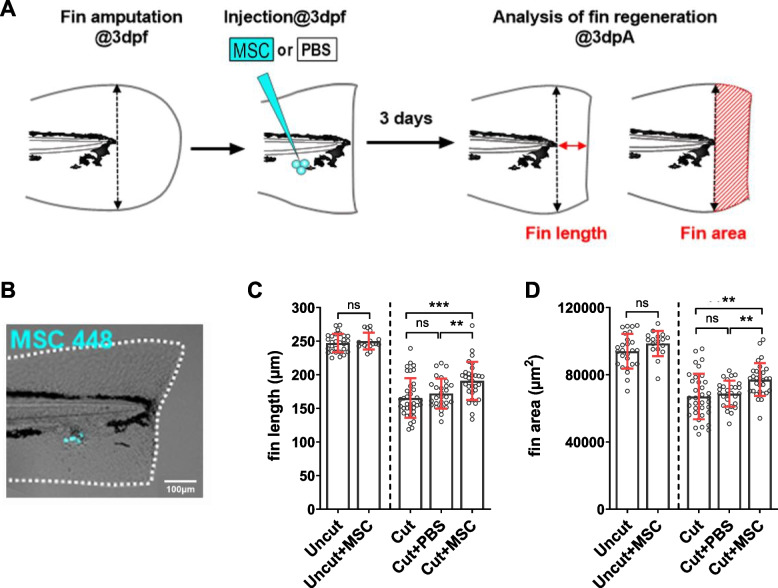
Exogenous murine MSCs enhance zebrafish fin regeneration. **A** Rationale of the regeneration assay. **B** Picture shows an example of successfully injected MSCs immediately after fin amputation at 3 dpf. (Scale bar = 100 µm). **C**-**D** Quantitative analysis of regeneration. Graphs show fin length (**C**) and area (**D**) at 3 dpA for Uncut control fins (with or without MSC injection) and cut control fins compared with MSC or PBS injected cut fins. Data are shown as mean ± SD. *n* = 26 (Uncut), 17 (Uncut + MSC), 35 (Cut), 28 (Cut + PBS), 30 (Cut + MSC). Statistical significance was assessed using non-parametric Mann–Whitney test. *: *p* < 0.05; **: *p* < 0.01; ***: *p* < 0.001; ****: *p* < 0.0001

To test the effects of MSCs in tissue regeneration, we assessed the impact of exogenous MSC grafts on caudal fin regeneration in zebrafish larvae (Fig. 1A). Immediately after fin amputation at 3 days post-fertilization (dpf), naïve murine MSCs (line MSC 448), previously isolated and characterized at the phenotypic and functional level by our laboratory (Luz-Crawford et al. 2015), were injected subcutaneously at the site of injury (Fig. 1A). Our observations indicated that the injected MSCs were not immediately rejected by the host, as they could still be detected in the regenerating fin at 4 h post-injection (Fig. 1B). Furthermore, compared to control conditions (amputation alone or amputation followed by PBS injection), MSC injection significantly enhanced fin regrowth at 3 days post-amputation (dpA). This regenerative effect was quantified by measuring the length and area of the regrown fin tissue from the amputation plane to the distal edge at 3 dpA (Fig. 1C-D). We detected no significant difference between both control conditions, suggesting that the injection by itself has no effect on fin regeneration. Notably, the pro-regenerative activity of exogenous MSCs was achieved with relatively low MSC numbers and was sufficient to accelerate the spontaneous regeneration process in zebrafish.

### Prostaglandin E2 mediates MSC-induced acceleration of caudal fin regeneration

It is now well accepted that protective functions of MSCs are mediated by secretion of soluble factors that enhance survival, proliferation and/or modulate the inflammatory response within the host tissue (Cosenza et al. 2017; Maumus et al. 2017; Maumus et al. 2013; Sarre et al. 2022). To test whether the pro-regenerative function of MSCs on zebrafish larvae after caudal fin amputation depends on common soluble factors derived from the grafted cells, we focused our attention on PGE2, a lipid signaling molecule with well documented pro-regenerative properties (Cheng et al. 2021), one of the major MSC-derived factors that have been linked to MSC immunoregulatory and therapeutic potential in different in vitro and in vivo models (Kota et al. 2017; Sareen et al. 2021; Wang et al. 2021).

**Fig. 2:**
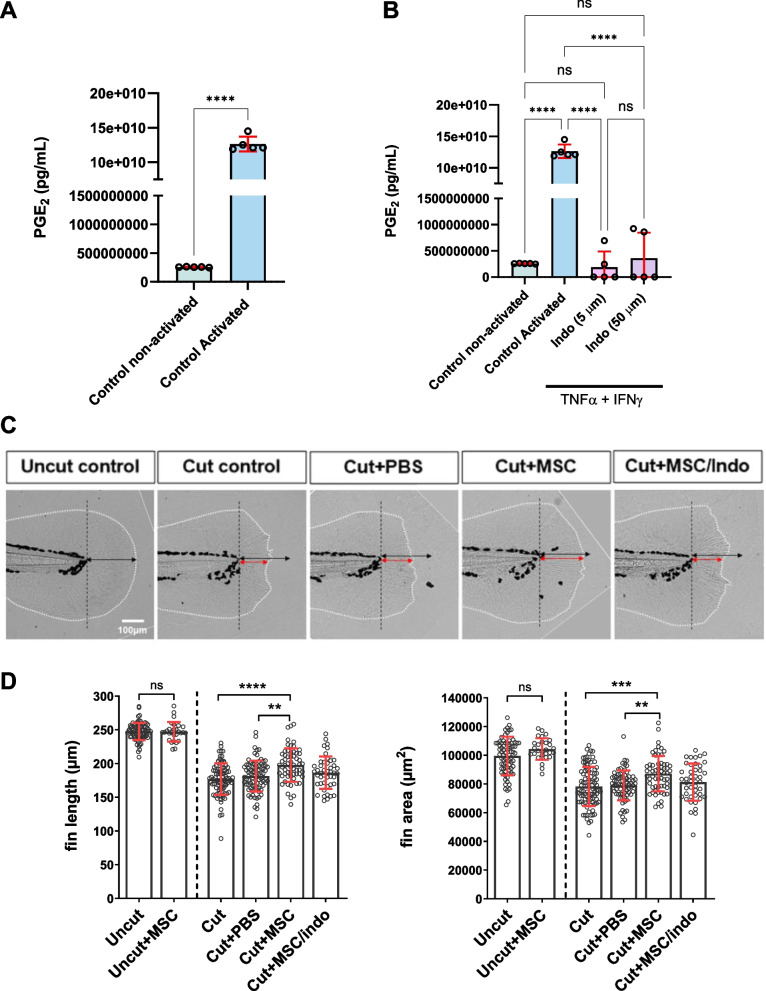
Indomethacin-treated MSCs exhibit reduced regenerative properties. **A** Quantification of PGE2 secretion measured by ELISA in MSCs under basal conditions (non-activated) or after stimulation with TNF-α and IFN-γ (activated). **B** Quantification of PGE2 secretion in activated MSCs incubated with a specific PGE2 inhibitor (indomethacin; Indo) at concentrations of 5 µM and 50 µM. **C**-**D** Quantitative analysis of fin regeneration. Graphs show fin length (**C**) and area (**D**) at 3 dpA for uncut fins (with and without cell injection), cut control fins and PBS or MSC-injected fins (either with naïve or indomethacin-pretreated MSC). Data are shown as mean ± SD. Sample sizes are as follows: ELISA assays (**A**-**B**), *n* = 5 independent experiments; regeneration assays (**C**-**D**), *n* = 78 Uncut, 92 Cut, 71Cut + PBS, 60 Cut + MSC, 44 Cut + MSC/Indo, 28 Uncut + MSC. Statistical significance was assessed using non-parametric Mann–Whitney test or one-way ANOVA with Dunn’s multiple comparisons. *p < 0.05 **: *p* < 0.01; ***: *p* < 0.001; ****: *p* < 0.0001

First, we quantified PGE2 secretion by MSCs under basal conditions (non-activated control) and following stimulation with TNF-α and IFN-γ, the main cytokines activating MSC immunoregulatory properties (Lopez-Garcia and Castro-Manrreza 2021). We found that PGE2 was minimally produced by non-activated MSCs, and that treatment with TNF-α and IFN-γ significantly increased its production compared to basal levels (Fig. 2A). The specificity of the inhibition of PGE2 secretion by indomethacin (Indo) treatment, a well-characterized inhibitor of cyclooxygenase enzymes (COX-1 and COX-2), which are essential for PGE2 biosynthesis, was confirmed at both concentrations tested (5 µM and 50 µM) (Fig. 2B).

To exclude the possibility that indomethacin treatment compromised MSC viability, we performed Annexin V/7-AAD flow cytometry and a Cell Death Detection ELISA after 24 h of treatment with 5 or 50 μM indomethacin, with or without TNFα/IFNγ activation (Fig. S1). No increase in apoptotic or dead cells was detected compared with untreated controls, demonstrating that indomethacin neither impaired MSC survival nor promoted apoptosis under our experimental conditions.

To investigate the role of PGE2 in MSC-mediated regeneration, we compared the effects of naïve MSCs and MSCs with impaired PGE2 production on zebrafish caudal fin regeneration. PGE2 synthesis was inhibited by pre-treating MSCs with indomethacin (5 µM) for 24 h. The regenerative capacity of zebrafish injected with naïve MSCs (cut + MSC) was subsequently compared to that of fish receiving indomethacin-pretreated MSCs (cut + MSC/Indo) (Fig. 2C-D). The beneficial effect observed with control MSCs was abolished when MSCs were pretreated with indomethacin (Fig. 2C-D). These results indicate that PGE2, a lipid mediator consistently implicated in the therapeutic functions of MSCs in mammals, also plays a critical role in the pro-regenerative effects of MSCs in zebrafish.

To further explore the possible contribution of endogenous zebrafish PGE2 production or signaling, we re-analyzed publicly available scRNA-seq datasets of regenerating caudal fins (Bohaud et al. 2022; Laplace-Builhe et al. 2021). UMAP clustering identified major blastema-associated populations, including fibroblasts, osteoprogenitors, macrophages, and epidermal/peridermal cells (Fig. S2A). Canonical biosynthetic genes, including *pla2g4aa, ptgs1, ptgs2a/b*, and *ptges/ptges3a/b*, were expressed primarily in epidermal/peridermal cells, with negligible expression in blastema fibroblasts, macrophages, and osteoprogenitors (Fig. S2B-C). Similarly, canonical PGE2 receptors (*ptger1a/b/c, ptger2a/b, ptger4a/b*) were barely detected across clusters (Fig. S3). In contrast, expression of PGE2 transporters (*slco2b1, abcc4*) and non-canonical mediators (*akt1, ctnnb1, mapk1, pparg*) was broadly distributed across blastema-associated cells (Fig. S4). These findings indicate that blastema cells lack an active endogenous PGE2 pathway, supporting the conclusion that exogenous MSCs represent the main functional source of PGE2 in this model.

### MSC-derived PGE2 regulates macrophage recruitment, activation, and MSC persistence during fin regeneration

**Fig. 3:**
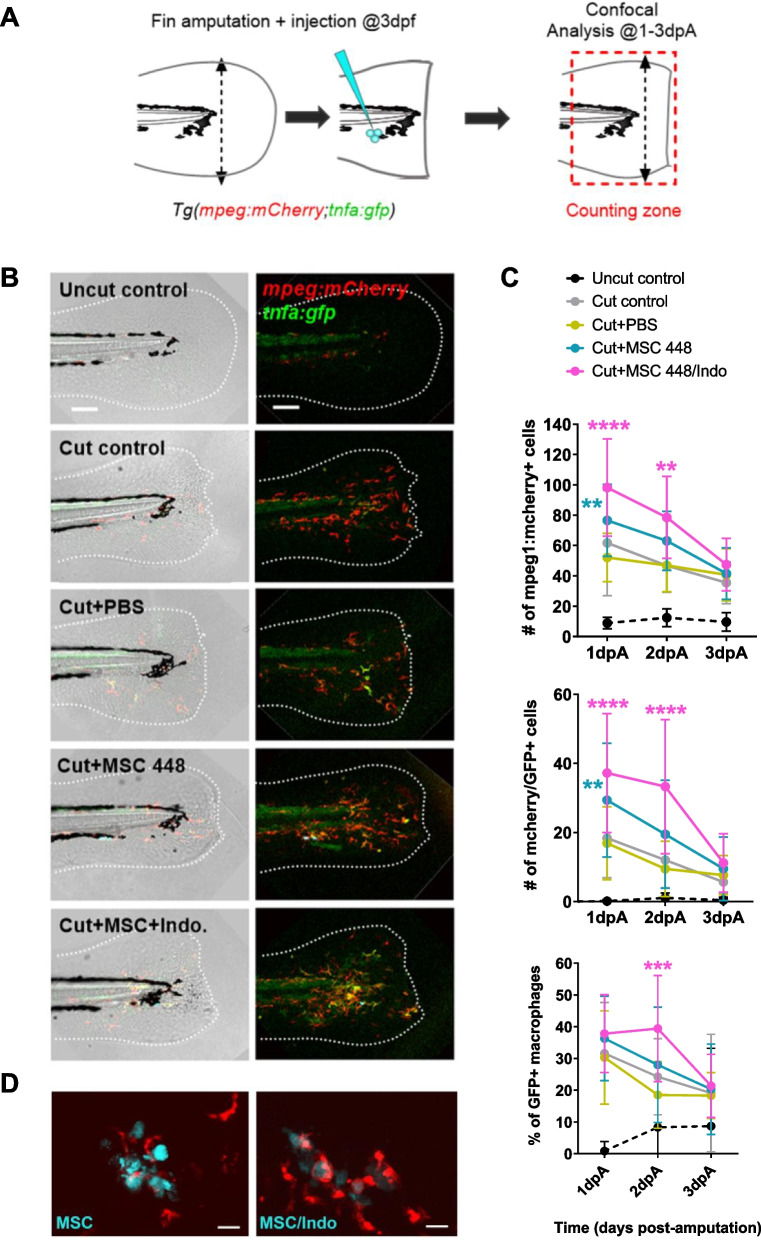
MSC-derived PGE2 regulates macrophage recruitment, inflammatory resolution, and MSC clearance during caudal fin regeneration. **A** Rationale of the experiment. **B** Confocal analysis of macrophage recruitment and polarization during fin regeneration at 48 hpA in *Tg(mpeg:mcherry/tnfa:gfp)*, one representative maximal projection of z-stack is given for each condition. Dotted white lines delineate the fin contours. **C** Quantification of total macrophages (mCherry +), pro-inflammatory macrophages (GFP +/mCherry +) and proportion of pro-inflammatory versus total macrophages during the time course of fin regeneration (1, 2 and 3 dpA). **D** Representative maximal projections of confocal z-stacks showing macrophage interactions with naïve or indomethacin-treated MSCs. Imaging was performed during a 3-h time-lapse sequence starting at 24 hpA after injection of exogenous MSCs (cyan) into *Tg*(*mpeg:mcherry*) larvae. Inhibition of PGE2 production increased MSC clearance by macrophages (red), as shown in the right panel (MSC/Indo) compared to the left panel (MSC). Data are shown as mean ± SD. *n* = 10 to 25 larvae per condition and time point. Statistical significance was assessed using two-way ANOVA followed by Sidak’s multiple comparisons. *: *p* < 0.05; **: *p* < 0.01; ***: *p* < 0.001; ****: *p* < 0.0001

PGE2 has been shown to modulate macrophage functions (for review, see Luque-Campos et al. 2021) and to mediate MSC-driven immunoregulation in several inflammatory contexts in both humans and mice (Bernardo and Fibbe 2013). Since macrophages play a key role in the regeneration process of the caudal fin (Nguyen-Chi et al. 2017), we hypothesized that MSCs could accelerate regeneration through the modulation of macrophage functions. To investigate the impact of MSCs on macrophage recruitment and activation in our model, we used the *Tg(mpeg1:mCherry-F/tnfa:eGFP-F)* reporter line in which total macrophages can be tracked with the mCherry tag and the pro-inflammatory macrophages expressing *tnfa* are labeled with both reporters (Fig. 3A-C). Amputated 3 dpf *Tg(mpeg1:mCherry-F/tnfa:eGFP-F)* larvae were injected with either naïve MSCs, MSCs pre-treated with indomethacin or PBS alone and analyzed by fluorescence microscopy at 1, 2, and 3 dpA (Fig. 3A-C) to quantify the numbers of each macrophage subpopulation. Both the total number of macrophages and the number of *tnfa*-expressing macrophages were significantly increased following MSC injections compared to uninjected and PBS-injected controls at 1 dpA (Fig. 3C). This result suggests that the graft of both naïve and pre-treated MSCs enhanced global macrophage recruitment and inflammatory response triggered by caudal fin amputation, at least at the earliest step of the regenerative process.

At later stages, our observations revealed that a higher number of total macrophages and *tnfa*-expressing macrophages is retained at the wound site at 2 dpA following injection of pre-treated MSCs compared with fish injected with naïve MSC or controls, while these numbers returned to control levels in all other conditions (Fig. 3B-C). Analysis of the proportion of tnfa + macrophages (Fig. 3B-C) present at different time points during regeneration further showed that only fins injected with indomethacin pre-treated MSCs display a higher proportion of *tnfa*-expressing macrophages at 2 dpA, suggesting that the balance between pro- and anti-inflammatory macrophages is disrupted when MSCs are treated with indomethacin to inhibit PGE2 production.

Together, these data demonstrate that MSCs directly influenced the kinetics of macrophage response during fin regeneration. Exogenous MSCs transiently enhanced recruitment of total macrophages, including *tnfa*-expressing cells, to the wound at 1 dpA; this response resolved by 2–3 dpA in control MSC-injected fins, whereas indomethacin-pretreated MSCs induced higher numbers and a greater proportion of tnfa + macrophages at 2 dpA. This increased but rapidly resolving inflammatory response in the case of naïve MSCs could account for the accelerated regenerative process. In contrast, the injection of MSCs treated with indomethacin to inhibit PGE2 production (Bouffi et al. 2010) significantly increased the number of both macrophage subsets. However, the proportion of tnfa + macrophages remained significantly higher in this group of larvae compared to the others at 2 dpA. Inflammation resolution in the indomethacin-treated MSC group was observed only at 3 dpA. This result indicates that while MSCs transiently enhance the recruitment and activation of macrophages, they also accelerate resolution of inflammation by promoting a rapid shift from *tnfa* + to *tnfa*- macrophages during regeneration, in a PGE2-dependent manner.

Given these alterations in macrophage behavior upon PGE2 inhibition, we next asked whether this dysregulated immune response could also impact MSC persistence in vivo. Treatments that enhance MSC survival rate upon in vivo injection improve their therapeutic properties (Park et al. 2020; Sarre et al. 2022). This led us to investigate whether a reduced persistence of the injected cells could contribute to the loss of MSC regenerative properties following indomethacin treatment, by limiting their interaction with the cells of the regenerating host tissues. To address this question, we performed a confocal analysis in real time to visualize the interactions between injected MSCs, and macrophages recruited to the wound site (Fig. 3D), with or without indomethacin pre-treatment. Time-lapse imaging over a 3-h period, starting at 24 h post amputation after the injection of exogenous MSCs into *Tg(mpeg:mCherry)* larvae, demonstrated that while naïve MSCs are maintained and make numerous contacts with recruited macrophages, MSCs treated with Indo were rapidly phagocytosed. Maximal projections of confocal z-stacks revealed differential macrophage behavior upon contact with naïve MSCs (Fig. S5) versus MSCs pre-treated with indomethacin. These findings suggest that the impaired regenerative potential of indomethacin-treated MSCs may be attributed to their accelerated clearance from the injury site, reducing their capacity to exert paracrine and immunomodulatory effects essential for tissue repair.

### PGE2 inhibition enhances MSC phagocytosis by activated murine macrophages: a conserved mechanism across species

**Fig. 4:**
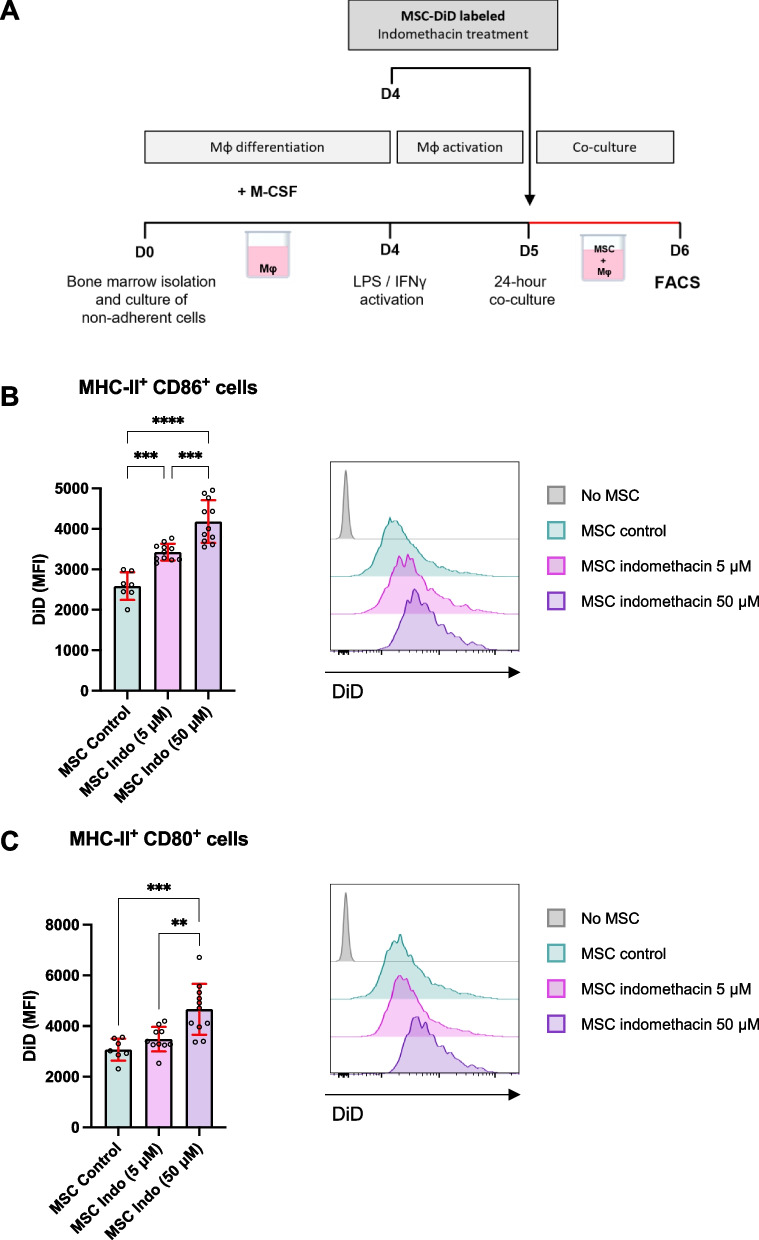
Murine MSCs treated with indomethacin are more susceptible to phagocytosis by activated primary macrophages. **A** Experimental design: bone marrow-derived monocytes were differentiated into macrophages (Mφ) with M-CSF. Macrophages were then activated with LPS + IFN-γ to induce a pro-inflammatory M1-like phenotype. Activated macrophages were co-cultured in direct contact with control or indomethacin-treated MSCs. Macrophage phenotype was analyzed by flow cytometry (FACS), with all cells gated on the CD45^+^CD11b^+^F4/80^+^ population, representing differentiated macrophages. **B**-**C** Median fluorescence intensity (MFI) of DiD, indicating phagocytosis, was measured in the pro-inflammatory macrophage subpopulations MHC-II^+^CD86^+^ (**B**) and MHC-II^+^CD80^+^ macrophages (**C**). Representative histograms are shown alongside MFI values. Data are shown as mean ± SD. *n* = 3 independent experiments. Statistical significance was assessed using one-way ANOVA. *: *p* < 0.05; **: *p* < 0.005; ***: *p* < 0.001; ****: *p* < 0.0001

To further investigate the role of PGE2 on mammalian MSC/macrophage interactions at cellular level, we examined the phagocytosis of indomethacin-treated MSCs (Indo-MSCs) by murine macrophages compared to control MSCs. Following 4 days of monocyte differentiation in the presence of M-CSF, macrophages were activated with LPS (250 ng/ml) and IFN-γ (20 ng/ml) for 24 h to upregulate MHC-II, CD80, and CD86 expression. Activated macrophages were then co-cultured for 24 h with DiD-labeled MSCs, which were either untreated (MSC control) or pre-treated with indomethacin at 5 or 50 μM (Fig. 4A). Flow cytometry analysis revealed that macrophages exhibited a significantly higher rate of phagocytosis of indomethacin-treated MSC compared to control MSC, with phagocytosis rates increasing proportionally to the concentration of indomethacin used (Fig. 4B-C). Notably, indomethacin-induced PGE2 inhibition enhanced the phagocytic activity of MHC-II^+^CD86^+^ and MHC-II^+^CD80^+^ macrophages towards MSCs. This effect was dose-dependent, varying according to the concentration of indomethacin applied to the MSCs (Fig. 4B-C). These findings suggest that indomethacin-mediated PGE2 inhibition in MSCs promotes their phagocytosis by activated macrophages, highlighting a conserved mechanism across mammalian and non-mammalian macrophages in response to MSCs administration.

## Discussion

This study highlights that zebrafish larva represents a powerful model for investigating the regenerative potential of MSCs and their interactions with the immune system in this context. By leveraging the zebrafish’s robust regenerative capacity, we explored how MSCs influence the kinetics of caudal fin regeneration and modulate macrophage recruitment, activation, and polarization. Importantly, our findings highlight the immunoregulatory potential of MSCs during regeneration and the crucial role of PGE2 as a mediator of these effects on zebrafish and murine macrophage behavior.

We demonstrated that pharmacological inhibition of PGE2 production by MSCs with indomethacin abolished the pro-regenerative effects of MSCs in zebrafish, establishing a causal role for this lipid mediator in MSC-driven tissue regeneration. When MSCs were treated with indomethacin to repress PGE2 production, their capacity to accelerate caudal fin regeneration was lost. This was associated with a stable accumulation of pro-inflammatory macrophages expressing *tnfa* at 1–2 dpA. In contrast, control MSCs enhanced fin fold regeneration by significantly increasing macrophages expressing *tnfa* at 1 dpA, then inducing a rapid and sustained polarization towards non-inflammatory states from 2 to 3 dpA. These findings show the critical interplay between MSCs and macrophages and indicate that MSC-derived PGE2 plays a key role in orchestrating macrophage plasticity, shifting the immune balance toward a pro-regenerative environment, in line with mammalian studies in which MSC-derived PGE2 has been implicated in tissue repair and immunomodulation (MacKenzie et al. 2013; Manferdini et al. 2017; Saleh et al. 2020; Vasandan et al. 2016; Wang et al. 2021).

Additionally, this study sheds light on the fate of MSCs in vivo, showing that MSCs treated with indomethacin were more susceptible to phagocytosis by both zebrafish and mammalian macrophages compared to untreated MSCs. This suggests that PGE2 not only mediates MSC immunoregulatory functions but may also influence their survival and persistence in the regenerative environment. Supporting this notion, several studies have shown that MSC-derived PGE2 acts in an autocrine manner to sustain proliferation, upregulate anti-apoptotic proteins such as BCL-2 and BCL-XL, and protect MSCs against apoptosis under stress conditions (Dhingra et al. 2013; Komatsu et al. 2020; Lee et al. 2016). The enhanced phagocytosis of indomethacin-treated MSCs observed here is therefore unlikely to result from drug-induced cytotoxicity, as confirmed by our viability assays, but rather from the loss of PGE2-dependent protective and immunoregulatory signals. A potential limitation of our study is that lipophilic dyes such as DiD may transfer between adjacent cells, raising the possibility of artefactual labeling. However, the consistent results obtained with the non-transferable cytoplasmic dye CTV. Together with direct time-lapse confocal visualization of the engulfment of treated MSC by macrophages in zebrafish, these data strongly support that the observed events represent direct macrophage-mediated phagocytosis of MSCs.

Complementing these findings, re-analysis of published scRNA-seq datasets from regenerating zebrafish fins (Laplace-Builhe et al. 2021) revealed negligible expression of key biosynthetic enzymes (*pla2g4aa, ptgs1/2, ptges, ptges3a/b*) and canonical PGE2 receptors in blastema fibroblasts, osteoprogenitors, and macrophages, while transporters and non-canonical mediators were broadly detected. These data suggest that endogenous blastema cells contribute little to PGE2 signaling, reinforcing the view that exogenous MSCs represent the primary functional source of PGE2 in this model. Nevertheless, we cannot fully exclude contributions from other tissues adjacent to the blastema, such as epidermis or vasculature, that may provide low levels of prostaglandins during regeneration. Future studies could address this possibility with functional approaches such as the use of zebrafish lines carrying targeted deletions of ptgs genes in specific cell types, which would provide a more definitive assessment of endogenous PGE2 production and its relevance for regeneration.

Taken together, our results reveal a dual role for MSC-derived PGE2: promoting MSC persistence in the regenerative niche and dynamically modulating macrophage behavior to accelerate inflammation resolution. The zebrafish model thus provides a unique platform to monitor MSC fate and immune interactions in vivo, bridging insights from mammalian studies to a regenerative context. Future work should study downstream mechanisms, including EP2/EP4–cAMP–PKA signaling, and employ genetic or pharmacological approaches to manipulate COX/PTGES pathways in specific cell types. In parallel, direct in vivo quantification of PGE2 production during regeneration will be essential to firmly establish its contribution to MSC therapeutic function. Overall, our findings emphasize that MSC therapeutic potential is closely linked to their capacity to produce PGE2, which safeguards both their survival and immunomodulatory capacity, thereby enabling efficient tissue regeneration.

Our work therefore gives evidence that the zebrafish larva is a valuable complementary model to mammalian systems, enabling high-resolution dissection of MSC–immune interactions during regeneration. By bridging insights across species, this model provides a unique preclinical platform to identify conserved mechanisms, such as PGE2-mediated immunoregulation, that could be harnessed to enhance MSC-based therapies in translational settings.

## Conclusions

These results provide a strong foundation for future efforts to optimize MSC-based therapies. Targeting the PGE2 axis within MSCs, either pharmacologically or genetically, could represent valuable strategies to modulate macrophage responses and enhance their regenerative potential in clinical applications. In this context, zebrafish offers a powerful model to test the efficacy of enhanced therapeutic cells and to better understand their mechanisms of action. Building on these findings, future studies may identify additional pathways and mechanisms that can be harnessed to improve tissue repair and regeneration across diverse settings.

## Methods

### Zebrafish

All experiments using animals were performed at the University of Montpellier according to the European Union guidelines and were approved by the *Comité d’éthique en expérimentation animale* n°36 (approval number: A3417237, reference: APAFIS #32511–2021072114172657 v2). Zebrafish lines were maintained as adults under standard conditions (Nguyen-Chi et al. 2014). Larvae were obtained by natural spawning from pairs of adults and raised at 28.5 °C. The zebrafish lines used in this study were: AB wild type zebrafish (ZIRC) for assessing fin regeneration and *Tg(mpeg1.1:mCherry-F)*^ump2Tg^ (herein abbreviated *Tg(mpeg:mCherry)*; Nguyen-Chi et al. 2015) to track in vivo macrophage behavior. *Tg(tnfa:eGFP-F)*^*ump5Tg*^ were crossed with *Tg(mpeg1.1:mCherry-F)*^ump2Tg^ to obtain double transgenics *Tg(mpeg1:mCherry-F/tnfa:eGFP-F)* (herein abbreviated *Tg(mpeg:mcherry/tnfa:gfp)*) that were used to distinguish *tnfa* expressing (pro-inflammatory) macrophage subset from total mCherry + macrophages.

### Caudal fin amputation in zebrafish larvae

Fin amputations were performed under a classical macroscope in 3 dpf larvae anesthetized with 0.016% Tricaine (MS222, Sigma) diluted in fish water. Fins were cut with sterile scalpel posterior to the notochord, removing approximately 70–80% of the total length of the fin. After amputation, larvae were either returned at 28.5 °C into a Petri dish containing fresh fish water to recover (“Cut control”) until the desired stages or processed immediately for further MSC- or PBS- injection (“Cut + MSC” or “Cut + PBS”). Uninjured age-matched larvae were only anaesthetized for the same period without amputation, and then either kept at 28.5 °C as “Uncut control”, or injected with MSC (“Uncut + MSC”).

### Murine mesenchymal stromal cell isolation and amplification

Murine MSCs were isolated from the bone marrow of 6–8-week-old C57BL/6 mice (Charles River, France) and characterized as previously described (Bouffi et al. 2010). Briefly, bone marrow was extracted by flushing the long bones. The resulting cell suspension was seeded and cultured in a complete medium containing high-glucose DMEM supplemented with 10% fetal bovine serum (FBS), 2 mM L-Glutamine, and 1% penicillin/streptomycin (Gibco, Thermo Fisher Scientific, USA). Non-adherent cells were removed after initial culture. MSCs were maintained at subconfluence and expanded at a density of 5,000 cells/cm^2^. Cells between passages 13 and 20 were used for subsequent experiments.

### MSC treatment

For in vivo injections and macrophage polarization experiments, MSCs were labeled with cell tracking dyes depending on the experimental context. For in vitro co-cultures with primary macrophages, MSCs were labeled with the lipophilic dye DiD (Invitrogen, USA) following the manufacturer’s protocol. For *in* vivo injections into zebrafish larvae, MSCs were labeled with the cytoplasmic tracker CellTrace Violet™ (CTV, Thermo Fisher Scientific, USA). In both cases, cells were incubated with the dye for 15 min at 37ºC in serum-free medium, followed by two washes with PBS before use.

For pharmacological inhibition, MSCs were treated with either 5 µM or 50 µM indomethacin (Sigma-Aldrich, USA) for 24 h at 37 °C in a humidified atmosphere containing 5% CO_2_. After treatment, cells were washed twice with PBS before being used for co-culture or injection experiments.

### PGE2 production by enzyme-linked immunosorbent assay

To assess PGE2 production by MSCs, cells were pre-treated with 5 µM or 50 µM indomethacin (Sigma-Aldrich, USA) for 1 h, followed by activation with recombinant murine IFN-γ (20 ng/ml) and TNF- α (10 ng/ml) (R&D Systems, USA) for a total of 24 h as previously described. Indomethacin was maintained in the culture medium throughout the activation period. After this time, supernatants were collected, and PGE2 concentration was quantified using a Prostaglandin E2 Enzyme Immunoassay kit (Arbor Assays, USA) according to the manufacturer’s instructions.

### MSC viability assessment

To assess whether indomethacin affected MSC viability, cells were pre-treated with 5 µM or 50 µM indomethacin as described above, followed by stimulation with recombinant murine IFN-γ (20 ng/ml) and TNF- α (10 ng/ml). As positive control of apoptosis, MSCs were treated with 20 µM menadione (MP Biomedicals, USA) for 30 min at 37 ºC prior to the assays. Cell viability was evaluated by Annexin V/7-AAD staining. Briefly, cells were harvested by trypsinization, washed once with annexin V binding buffer (BD Biosciences, USA), and incubated with Annexin V-FITC (BD Biosciences, USA) for 20 min at 4 ºC. After one wash, cells were labeled with 7-AAD for 15 min at 4ºC and immediately analyzed using a Cytek Aurora spectral flow cytometer, with SpectroFlo**®** software (Cytek Bioscience, USA). In parallel, viability was also assessed using the Cell Death Detection ELISA^PLUS^ kit (Roche, Switzerland), which quantifies cytoplasmic histone-associated DNA fragments as a hallmark of apoptosis. The assay was performed according to the manufacturer’s instructions.

### Murine macrophage polarization

Bone marrow-derived monocytes were isolated from C57BL/6 mice through femoral flushing to obtain a single-cell suspension. Cells were cultured in 100 mm plates in Mixed Lymphocyte Reaction (MLR) medium, consisting of Iscove’s Modified Dulbecco’s Medium (IMDM) supplemented with 10% heat-inactivated FBS, 20 mM HEPES, 0.1 mM non-essential amino acids, 2 mM L-Glutamine, 1 mM sodium pyruvate, 100 U/ml penicillin, 100 µg/mL streptomycin (Gibco, USA), and 50 µM β-mercaptoethanol (Life Technologies, USA). After 4 h of incubation at 37 °C in a humidified 5% CO_2_ atmosphere, non-adherent cells were collected and reseeded in multi-well plates at a density of 2 × 10^5^ cells/cm^2^. To drive macrophage differentiation, MLR medium was supplemented with 20 ng/ml macrophage colony-stimulating factor (M-CSF) (Miltenyi Biotec, Germany) on days 0, 1, and 4. On day 5, macrophages were pre-activated for 24 h using 250 ng/ml lipopolysaccharide (LPS) (Sigma-Aldrich, USA) and 20 ng/ml interferon-γ (IFN-γ) (R&D Systems, USA). On day 6, the activated macrophages were co-cultured in direct contact with either control or indomethacin-treated MSCs at a 1:10 ratio (MSC:Macrophages) in MLR medium. Co-cultures were maintained for 24 h at 37 °C in a humidified 5% CO_2_ atmosphere before downstream analysis.

### Flow cytometry

Macrophage polarization was assessed using flow cytometry. Cells were harvested with Versene (Gibco, USA) and incubated with Fc block (BD Biosciences, USA) according to the manufacturer’s instructions. Cell viability was determined using the LIVE/DEAD™ Near-IR fixable viability dye (Life Technologies, USA). Subsequently, cells were stained with specific antibodies targeting CD45 (clone 30-F11), CD11b (clone M1/70), CD80 (clone 16-10A1) (BD Biosciences), MHC-II (clone M5/114.15.2) (Miltenyi Biotec, Germany), and F4/80 (clone BM8) (eBioscience, USA). Flow cytometric analyses were performed using a BD FACSymphony A5 Flow Cytometer (BD Biosciences, USA) equipped with BD FACSDiva software (version 9.0). The collected data were analyzed with FlowJo software (version 10) (TreeStar Inc., USA).

### MSC/PBS injections

After fin amputation, 3 dpf anaesthetized larvae were mounted in 1.5% low-melting-point agarose (LMP agarose, Sigma Aldrich). CTV-labelled MSCs resuspended in sterile PBS were loaded in a glass capillary and ~ 2–4 nl were injected subcutaneously at the ventral side of the fin using a FemtoJet**®** pressure microinjector (Eppendorf, Germany). The fish that were considered successfully injected based on the presence CTV^+^ cells in their fin 2–4 h after injection, were selected for further analysis at 1, 2 or 3 days post-Amputation (dpA).

### Imaging

Confocal imaging of the uncut and regenerating fins was performed in live anesthetized larvae using Leica TCS SP5 and Leica TCS SP8 confocal microscopes (Leica Application Suite V3.2 and V3.5, respectively). Images were then analyzed using Fiji Software (ImageJ 1.52p). For time-lapse imaging, fish were mounted in 1% LMP agarose and covered with zebrafish water containing 0.016% Tricaine in 35 mm glass-bottom dishes sealed with parafilm to prevent evaporation.

### Cell counting

Macrophage quantification was done on confocal z-stacks of the uncut and cut fins of *Tg(mpeg:mcherry/tnfa:gfp)* larvae taken at 1, 2 and 3 dpA. The number of *mCherry* + and double *mCherry/GFP* + cells detected between the caudal vein/artery loop and the tip of the uncut or regenerating fin was then manually counted using Fiji software to mark the cells.

### Single-cell RNA sequencing data analysis

Publicly available single-cell RNA sequencing (scRNA-seq) data from regenerating zebrafish caudal fins were re-analyzed to investigate the expression of genes related to PGE2 biosynthesis and signaling. The datasets were originally generated and described in detail in previous publications from our group (Laplace-Builhe et al. 2021). Briefly, caudal fins were collected at different timepoints after amputation, dissociated, and subjected to droplet-based scRNA-seq (10x Genomics platform). Data preprocessing, quality control, clustering, and annotation of cell types were performed as described in the original studies.

For the present study, we specifically interrogated the expression of genes associated with PGE2 synthesis (*pla2g4aa, ptgs1, ptgs2a/b, ptges, ptges3a/b*), canonical receptors (*ptger1a/b/c, ptger2a/b, ptger4a/b*), transporters (*slco2b1, abcc4*) and non-canonical mediators potentially linked to PGE2 activity (*pparg, ctnnb1, pik3ca, akt1, mapk1*). Expression levels were visualized by feature plots, dot plots, violin plots, and UMAP embeddings of annotated blastema cell clusters.

### Statistical analysis

Details regarding the statistical analyses, including the tests used and significance levels, are provided in the legends of the corresponding figures.

## Supplementary Information

Supplementary Material 1: Supplementary Figure 1. Indomethacin treatment does not impair MSC viability. (A) Representative Annexin V/7-AAD dot plots of untreated MSCs, indomethacin-treated MSCs (5 or 50 μM), and positive control (menadione, 20 μM). (B) Quantification of early apoptotic (Annexin V^+^/7-AAD^-^) and late apoptotic/necrotic (Annexin V^+^/7-AAD^+^) MSCs after treatment with indomethacin, in the absence or presence of TNFα/IFNγ activation. (C) Cell Death Detection ELISA measuring DNA fragmentation in MSCs treated as in (B). Results are expressed as fold-change relative to the untreated control condition. Data are shown as mean ± SD, *n* = 4 independent experiments. Statistical significance was assessed using one-way ANOVA. **: *p* < 0.005; ****: *p* < 0.0001. Supplementary Figure 2. Expression of canonical PGE2 pathway genes in regenerating zebrafish fins. (A) UMAP visualization of major blastema-associated cell populations at 3 dpA. (B) Expression of the phospholipase A2 gene *pla2g4aa*, which catalyzes the release of arachidonic acid, was largely restricted to epidermal/periderm cells. (C) Expression of cyclooxygenase family genes (*ptgs1, ptgs2a, ptgs2b*) was minimal across blastema cell clusters. (D) Expression of PGE2 synthases (*ptges, ptges3a, ptges3b*) was negligible in fibroblasts, osteoprogenitors, and macrophages. Both UMAP feature plots and violin plots are shown for each gene. Representative plots are from re-analysis of published scRNA-seq data (Laplace-Builhe et al. 2021); no quantitative statistical analysis was performed. Supplementary Figure 3. Expression of canonical receptors in regenerating fins. UMAP and violin plots showing expression of canonical PGE2 receptors (*ptger1a, ptger1b, ptger1c, ptger2a, ptger2b, ptger4a, ptger4b*) across blastema-associated clusters at 3 dpA. Overall, receptor expression was negligible across fibroblasts, macrophages, and osteoprogenitors. Representative plots are from re-analysis of published scRNA-seq data (Laplace-Builhe et al. 2021); no quantitative statistical analysis was performed. Supplementary Figure 4. Expression of PGE2 transporters and non-canonical mediators in regenerating fins. (A) UMAP feature plots and violin plots showing expression of the PGE2 transporters *slco2b1* and *abcc4.* (B) Expression of alternative mediators and downstream signaling genes, including *akt1, ctnnb1, mapk1,* and *pparg,* which were broadly detected across blastema-associated cell subsets. Representative plots are from re-analysis of published scRNA-seq data (Laplace-Builhe et al. 2021); no quantitative statistical analysis was performed. Supplementary Figure 5. Extended visualization of MSC-macrophage interaction in vivo. (A) Orthogonal projections of z-stacks showing extensive cell–cell contacts between macrophages and MSCs. YZ and XZ panels show the proximity of macrophage prolongments with MSCs at two different time points during a time lapse analysis, indicated by arrowheads. (B) Maximal projections of z-stacks showing macrophages engulfing indomethacin-treated MSCs at three different time points during a 3-h time lapse acquisition. Arrowheads indicate engulfment events or close membrane apposition between macrophages and MSCs. No quantitative statistical analysis was performed.

## Data Availability

The datasets used and/or analyzed during the current study to support the conclusions are included in the article and in the corresponding additional files. They can be made available from the corresponding author on reasonable request.

## Abbreviations

MSCs: Mesenchymal stromal cells
PGE2: Prostaglandin E2
dpA: Days post-amputation
dpf: Days post-fertilization
COX: Cyclooxygenase enzymes
Indo: Indomethacin
PBS: Phosphate buffered saline
TNF: Tumor necrosis factor
LPS: Lipopolysaccharides
IFN: Interferon
FBS: Fetal bovine serum
DMEM: Dulbecco’s Modified Eagle Medium
CTV: CellTrace Violet
MFI: Mean of Fluorescence Intensity
