# Supplementary material for: Mammalian mesenchymal stromal cells enhance zebrafish fin regeneration
Source: Cell Regen. 2026 Jan 15;15:1. doi: 10.1186/s13619-025-00273-7 (PMC12808003; doi:10.1186/s13619-025-00273-7)
Supplement: Supplementary file 1 — Supplementary Material 1: Supplementary Figure 1. Indomethacin treatment does not impair MSC viability. (A) Representative Annexin V/7-AAD dot plots of untreated MSCs, indomethacin-treated MSCs (5 or 50 μM), and positive control (menadione, 20 μM). (B) Quantification of early apoptotic (Annexin V+/7-AAD-) and late apoptotic/necrotic (Annexin V+/7-AAD+) MSCs after treatment with indomethacin, in the absence or presence of TNFα/IFNγ activation. (C) Cell Death Detection ELISA measuring DNA fragmentation in MSCs treated as in (B). Results are expressed as fold-change relative to the untreated control condition. Data are shown as mean ± SD, n = 4 independent experiments. Statistical significance was assessed using one-way ANOVA. **: p < 0.005; ****: p < 0.0001. Supplementary Figure 2. Expression of canonical PGE2 pathway genes in regenerating zebrafish fins. (A) UMAP visualization of major blastema-associated cell populations at 3 dpA. (B) Expression of the phospholipase A2 gene pla2g4aa, which catalyzes the release of arachidonic acid, was largely restricted to epidermal/periderm cells. (C) Expression of cyclooxygenase family genes (ptgs1, ptgs2a, ptgs2b) was minimal across blastema cell clusters. (D) Expression of PGE2 synthases (ptges, ptges3a, ptges3b) was negligible in fibroblasts, osteoprogenitors, and macrophages. Both UMAP feature plots and violin plots are shown for each gene. Representative plots are from re-analysis of published scRNA-seq data (Laplace-Builhe et al. 2021); no quantitative statistical analysis was performed. Supplementary Figure 3. Expression of canonical receptors in regenerating fins. UMAP and violin plots showing expression of canonical PGE2 receptors (ptger1a, ptger1b, ptger1c, ptger2a, ptger2b, ptger4a, ptger4b) across blastema-associated clusters at 3 dpA. Overall, receptor expression was negligible across fibroblasts, macrophages, and osteoprogenitors. Representative plots are from re-analysis of published scRNA-seq data (Laplace-Builhe [file 13619_2025_273_MOESM1_ESM.pdf]

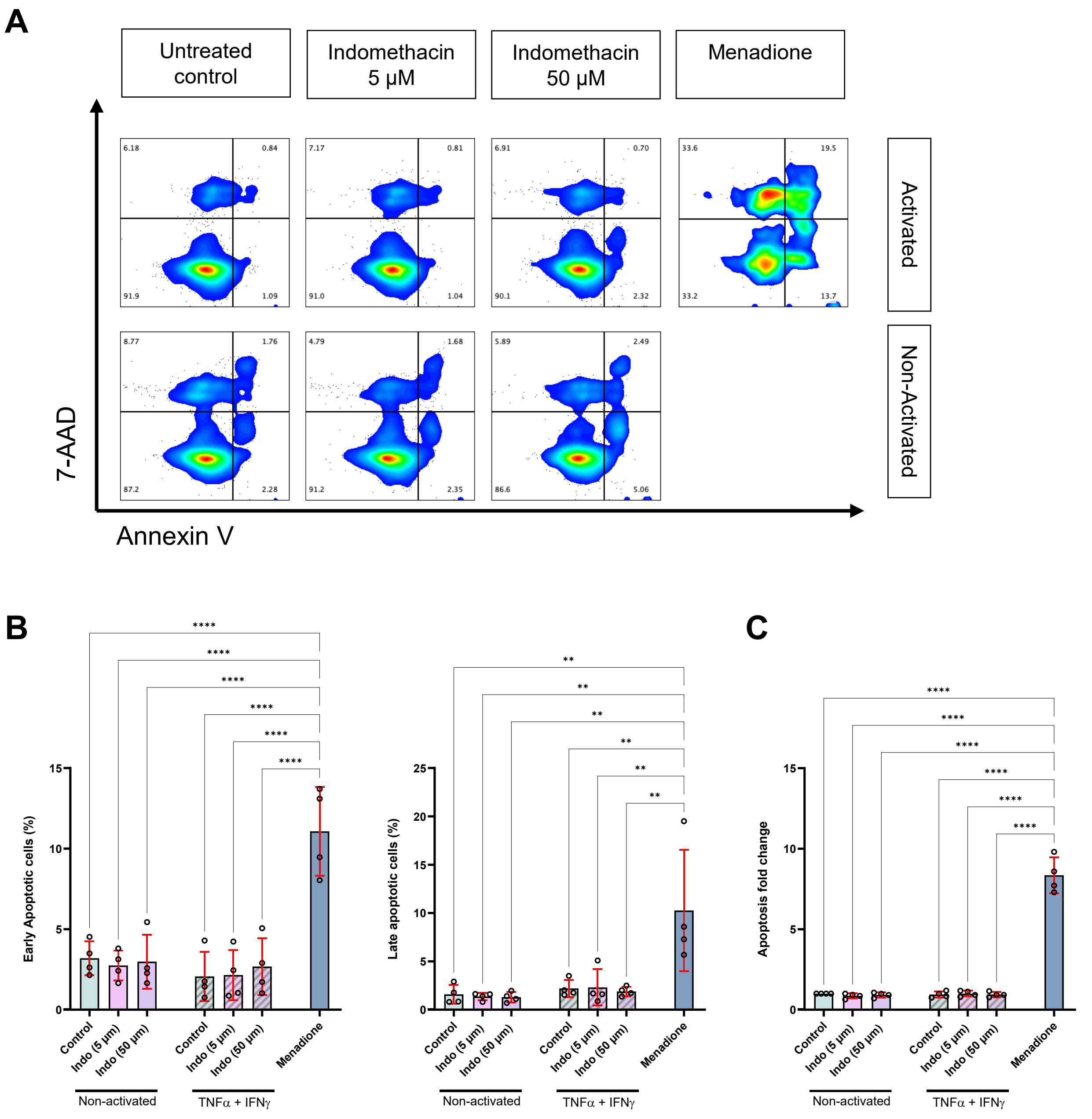

Supplementary Figure 1

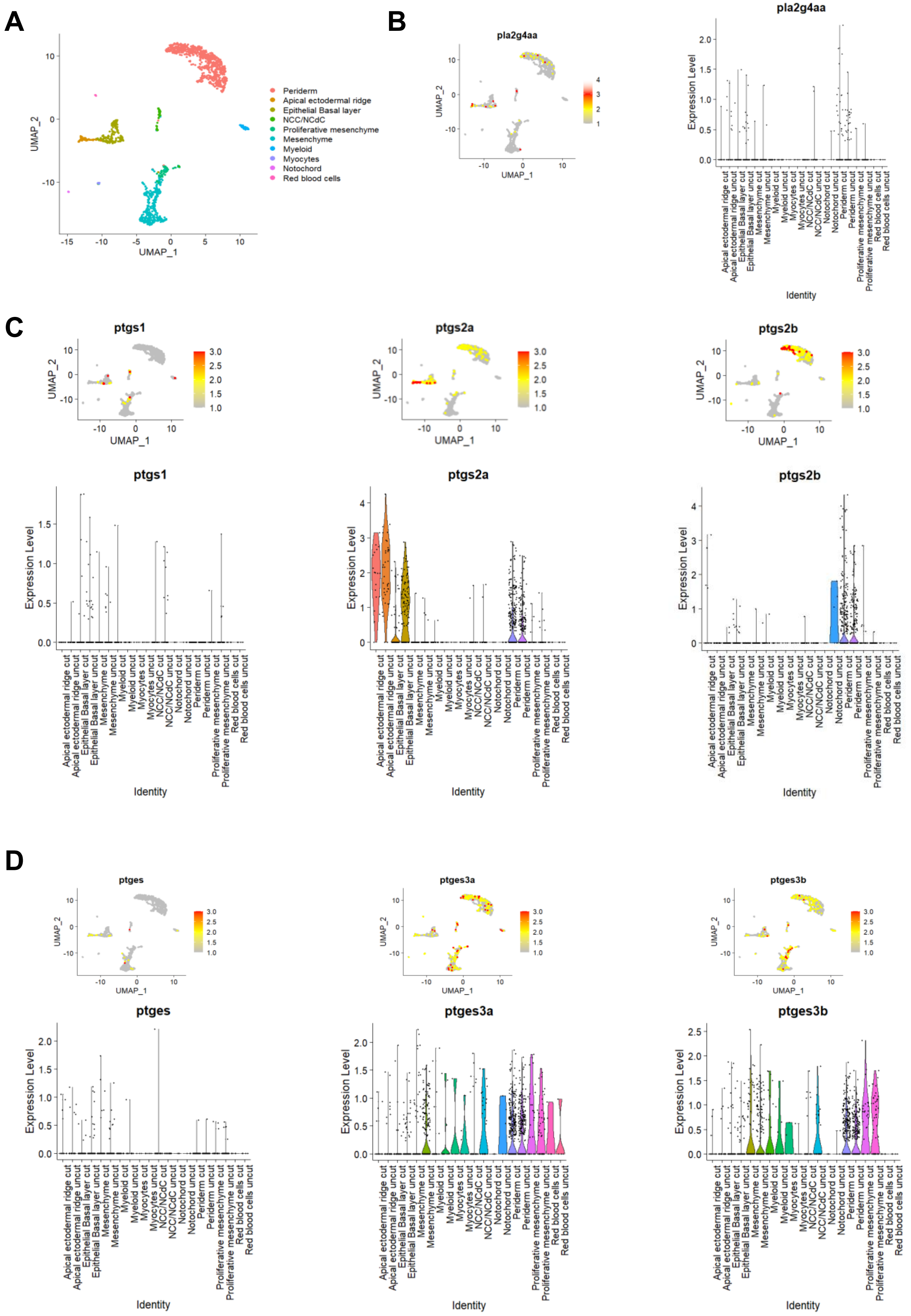

Supplementary Figure 2

A

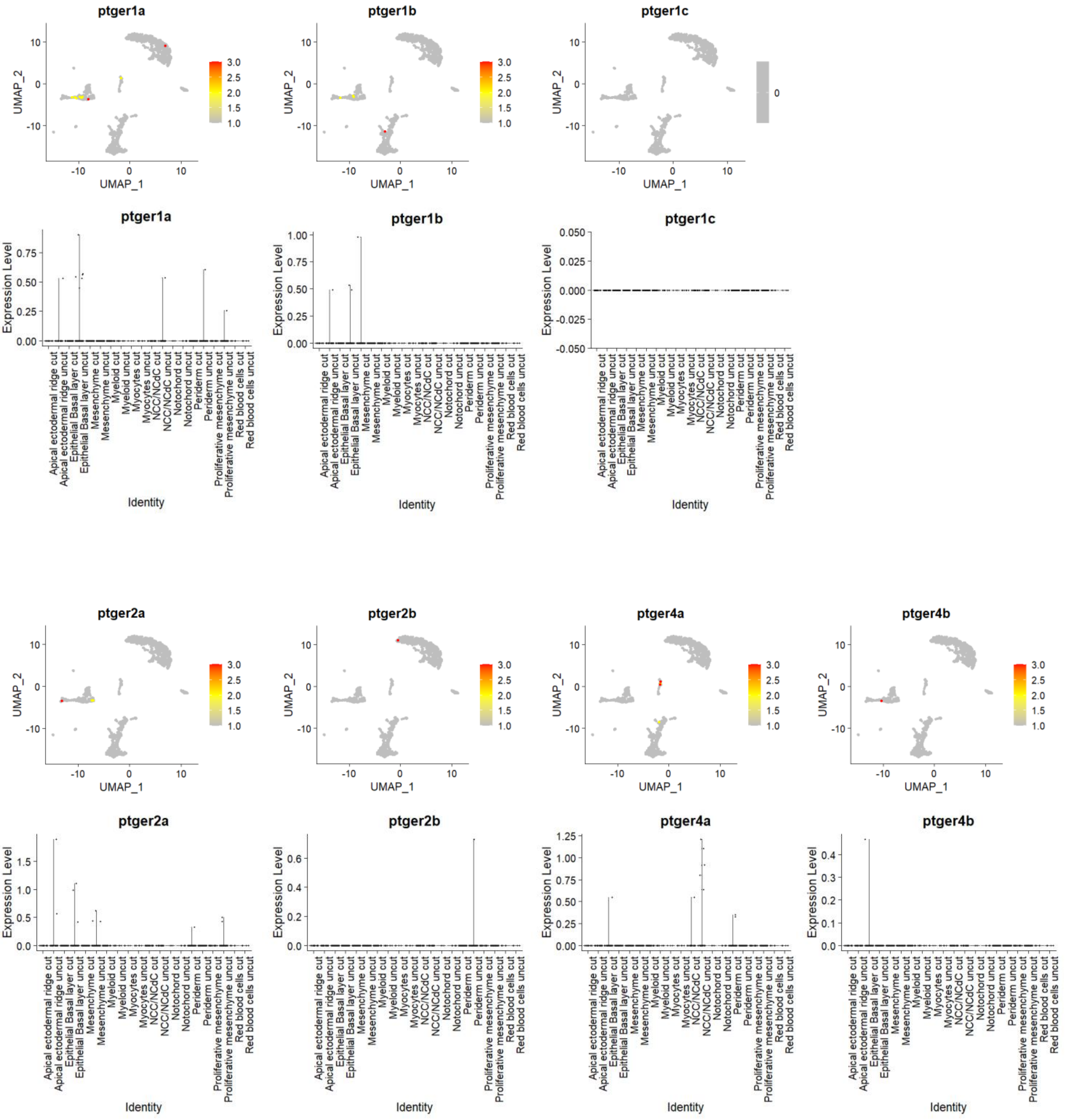

Supplementary Figure 3

A

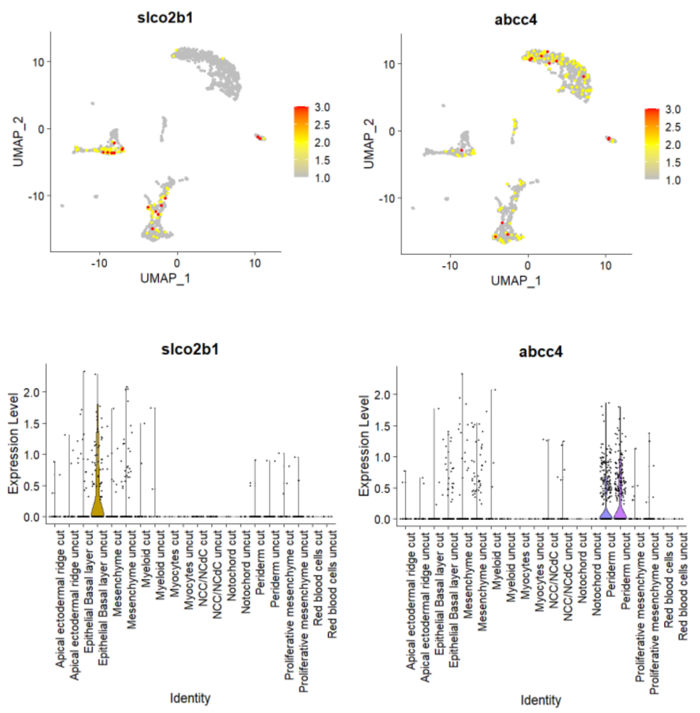

B

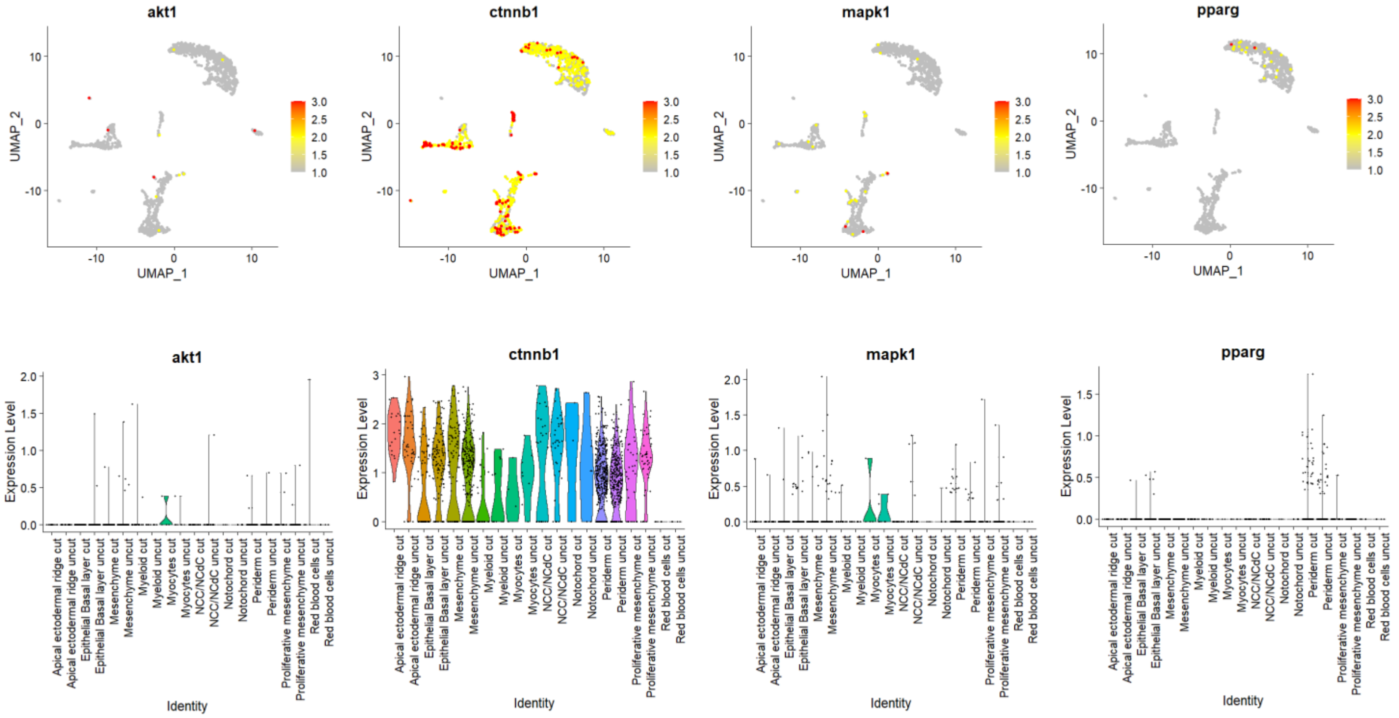

Supplementary Figure 4

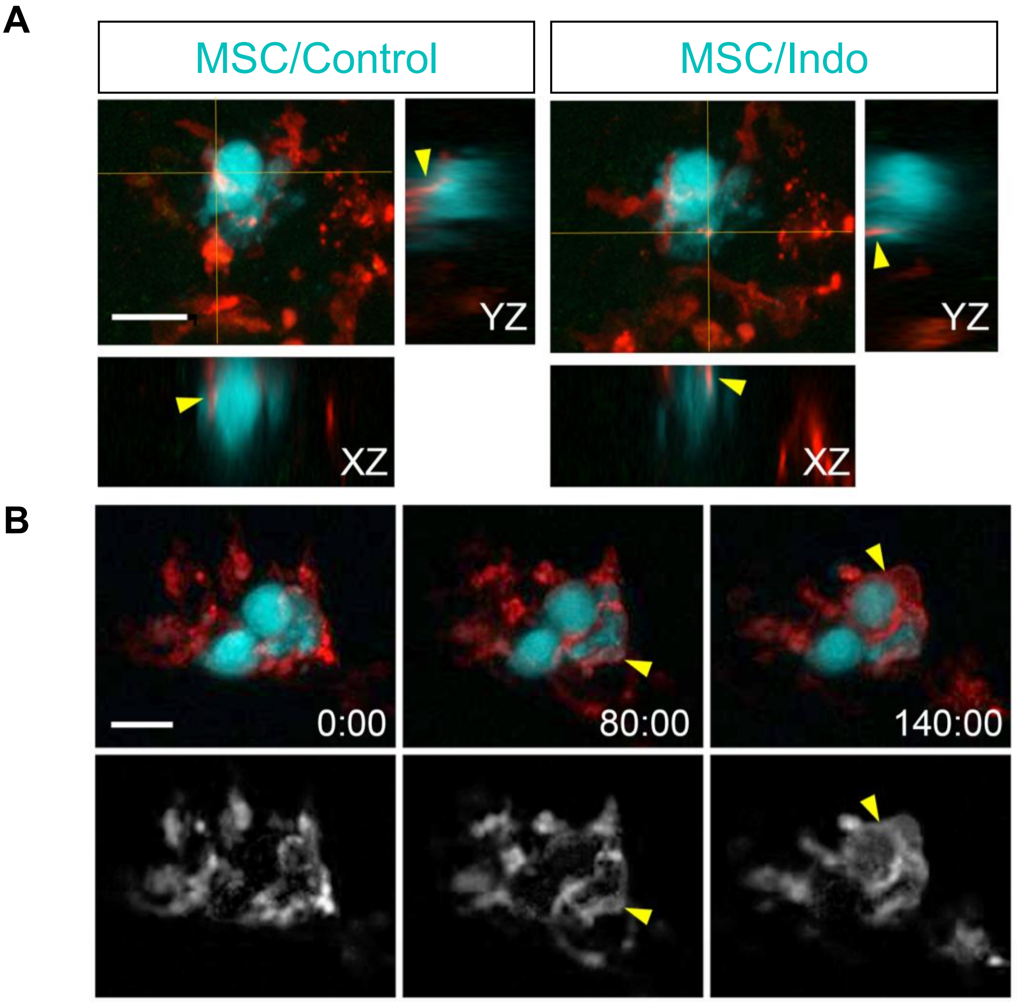

Supplementary Figure 5
